# Cardia Gastric Cancer Is Associated With Increased PIK3CA Amplifications and HER2 Expression Than Noncardia Gastric Cancer According to Lauren Classification

**DOI:** 10.3389/fonc.2021.632609

**Published:** 2021-06-08

**Authors:** Shih-Min Pai, Kuo-Hung Huang, Ming-Huang Chen, Wen-Liang Fang, Yee Chao, Su-Shun Lo, Anna Fen-Yau Li, Chew-Wun Wu, Yi-Ming Shyr

**Affiliations:** ^1^ Division of General Surgery, Department of Surgery, Taipei Veterans General Hospital, Taipei, Taiwan; ^2^ School of Medicine, National Yang Ming Chiao Tung University, Taipei, Taiwan; ^3^ Center of Immuno-Oncology, Department of Oncology, Taipei Veterans General Hospital, Taipei, Taiwan; ^4^ Department of Surgery, National Yang Ming Chiao Tung University Hospital, Yilan, Taiwan; ^5^ Department of Pathology, Taipei Veterans General Hospital, Taipei, Taiwan

**Keywords:** gastric cancer, cardia, noncardia, genetic alteration, *PIK3CA* amplification, *HER2* expression

## Abstract

**Background:**

To date, few reports have investigated genetic alterations and clinicopathological features in cardia and noncardia gastric cancer (GC).

**Methods:**

In total, 435 GC patients receiving curative surgery were included. The clinicopathological features, recurrence patterns, prognoses and genetic alterations were compared between cardia and noncardia GC patients.

**Results:**

Among the 435 enrolled patients, 47 (10.8%) had cardia GC. Compared with noncardia GC, cardia GC was associated with more intestinal-type tumors and similar initial recurrence patterns and 5-year overall survival (OS; 50.8% vs. 50.5%, *P* = 0.480) and disease-free survival (DFS; 48.6% vs. 48.9%, *P* = 0.392) rates. For both intestinal-type GC and diffuse-type GC, the clinicopathological features and 5-year OS and DFS rates were not significantly different between the cardia and noncardia GC patients. Multivariable analysis showed that cardia GC was not an independent prognostic factor. Compared with noncardia GC, cardia GC was associated with increased *PIK3CA* amplification than in patients with intestinal-type GC and was associated with increased *HER2* expression in patients with diffuse-type GC.

**Conclusions:**

Cardia GC is not an independent prognostic factor. In cardia GC patients with intestinal-type GC, *PIK3CA* amplification was more common, and in those with diffuse-type GC, *HER2* expression was more common. Targeted therapy may be beneficial for these patient subgroups.

## Introduction

Gastric cancer (GC) is the sixth most common cancer and ranks second in terms of cancer-related death worldwide (1). The incidence of cardia GC has shown an increasing trend over time, while that of noncardia GC has shown a decreasing trend.

According to the 8th edition of the American Joint Committee on Cancer (AJCC)/Union for International Cancer Control (UICC) tumor, node, metastasis (TNM) classification system (2), Siewert type 2 cardia cancer with esophagogasric junction invasion is classified as esophageal cancer, and Siewert type 3 cardia cancer is classified as GC. It was reported that cardia GC was associated with more advanced staging and unfavorable clinicopathological features at diagnosis than noncardia GC (3).

In terms of molecular analyses, some reports have investigated genetic alterations in cardia and noncardia GC (4, 5). Genome-wide association studies have revealed that some single nucleotide polymorphisms (SNPs) are associated with noncardia GC only (4), while others have shown no difference in the SNPs between cardia and noncardia GC (5). In addition, *HER2* expression has been reported to be higher in cardia GC than in noncardia GC (6). The correlation between *HER2* expression and histologic types in GC is controversial; *HER2* expression has been reported to be associated with intestinal-type GC in some studies (7), whereas other studies have indicated that *HER2* expression was more common in diffuse-type GC (8). Consequently, there is a need to investigate the correlation between genetic alterations and histologic type in cardia and noncardia GC.

The aim of the current study was to compare the clinicopathological characteristics, recurrence patterns, and prognoses between cardia and noncardia GC after curative surgery. As in a previous study (9), a nine-gene mutation panel was used to perform genetic analysis with the MassARRAY method; in addition, *Helicobacter pylori* (HP) infection, Epstein–Barr virus (EBV) infection, and *PIK3CA* amplification were compared between cardia and noncardia GC patients.

## Materials and Methods

Between 2005 and 2014, a total of 435 GC patients with adenocarcinoma who underwent curative surgery were included in the present study. According to the 8^th^ edition of the AJCC/UICC TNM classification system, among the 435 GC patients, 47 (10.8%) patients were classified as having cardia GC. Tumor and normal gastric mucosa tissues were collected, and genetic alterations were analyzed for all 435 GC patients. The Institutional Review Board of Taipei Veterans General Hospital approved the present study (2020-09-017CC). All samples used in this study had been previously collected from the biobank of Taipei Veterans General Hospital and were anonymized. All enrolled patients signed an informed consent form before sample collection from the biobank.

Regarding the extent of lymphadenectomy, at least D1+ dissection was performed for early GC, while D2 dissection was performed for advanced GC (10).

The follow-up examinations were the same as those performed in a previous study (11) and were conducted every 3 months during the first 3 years after surgery and every 6 months thereafter. Patients with tumor recurrence could receive 5-fluorouracil (FU)-based chemotherapy. No patients enrolled in the present study received preoperative chemotherapy. Adjuvant therapy, such as S-1, has been prescribed for stage II or III disease after curative surgery at our institute since 2008 due to its proven survival benefit (12). Approximately 62% of the enrolled patients underwent surgery before 2008. Consequently, the number of patients who received adjuvant chemotherapy was low in the present study.

### Analysis of Microsatellite Instability and Genetic Mutations

As reported in a previous study (13), five reference microsatellite markers were used to determine microsatellite instability (MSI), namely, D5S345, D2S123, D17S250, BAT25 and BAT26. MSI-high (MSI-H) was defined as ≥2 loci of instability, while MSI-low/stable (MSI-L/S) was defined as one locus or no MSI loci of instability (13).

As demonstrated in a previous study (9), a MassARRAY system (Agena, San Diego, CA) was used to identify 76 mutation hotspots in nine GC-related genes: *PIK3CA*, *AKT1*, *AKT2*, *AKT3*, *PTEN*, *ARID1A*, *TP53*, *BRAF*, and *KRAS*. Mutations in *PTEN*, *PIK3CA*, *AKT1*, *AKT2*, or *AKT3* were defined as *PI3K/AKT* pathway genetic mutations.

### HP and EBV Detection

As mentioned in a previous study, HP infection was identified using polymerase chain reaction (PCR) assays (14).

EBV infection was identified using the *in situ* hybridization (ISH) technique for the detection of EBV-encoded small RNAs in formalin-fixed paraffin-embedded tissue samples (15).

### Immunohistochemical (IHC) Staining for HER2

*HER2* IHC staining was performed with anti-human c-erbB-2 A0485 polyclonal antibody (dilution 1:500; Dako) (16). An IHC score of 3+ or an IHC score of 2+ with a positive fluorescence *in situ* hybridization result was defined as positive *HER2* IHC staining.

### Statistical Analysis

IBM SPSS Statistics 25.0 (IBM Corp., Armonk, NY, USA) was used for statistical analyses. Categorical data were compared between groups using the χ^2^ test with Yate’s correction or Fisher’s exact test. Overall survival (OS) was defined from the date of surgery to the date of death or the last follow-up. Disease-free survival (DFS) was defined as the length of time after surgery during which the patient was alive without GC recurrence. The Kaplan–Meier method was applied for the survival analysis of OS and DFS. Multivariable analysis with Cox proportional hazards models was applied to analyze the independent prognostic factors of OS. Statistical significance was defined as a *P* value less than 0.05.

## Results

### Clinicopathological Features

Among the 435 GC patients, 47 (10.8%) had cardia GC. Regarding the clinicopathological characteristics, as shown in **Table 1**, patients with cardia GC had more intestinal-type tumors than those with noncardia GC.

**Table 1:** Clinical profile between cardia and non-cardia GC patients.

	Non-cardia	Cardia	*P* value
	n = 388	n = 47	
	n (%)	n (%)	
Age (years old)			0.749
<65	158 (40.7)	18 (38.3)	
≧65	230 (59.3)	29 (61.7)	
Gender			0.535
Male	272 (70.1)	35 (74.5)	
Female	116 (29.9)	12 (25.5)	
Tumor size (cm)			0.630
<5	151 (38.9)	20 (42.6)	
≧5	237 (61.1)	27 (57.4)	
Extent of lymphadenectomy			0.721
D1+	98 (25.3)	13 (27.7)	
D2	290 (74.7)	34 (72.3)	
Gross appearance			0.220
Superficial type	58 (14.9)	6 (12.8)	
Borrmann types 1 & 2	102 (26.3)	18 (38.3)	
Borrmann types 3 & 4	228 (58.8)	23 (48.9)	
Lauren’s classification			**0.041**
Intestinal-type	203 (52.3)	32 (68.1)	
Diffuse-type	185 (47.7)	15 (31.9)	
Adjuvant chemotherapy	54 (13.9)	9 (19.1)	0.336
Lymphovascular invasion	275 (70.9)	32 (68.1)	0.692
Pathological T category			0.739
T1	59 (15.2)	8 (17.0)	
T2	66 (17.0)	5 (10.6)	
T3	132 (34.0)	17 (36.2)	
T4	131 (33.8)	17 (36.2)	
Pathological N category			0.404
N0	124 (32.0)	12 (25.5)	
N1	62 (16.0)	12 (25.5)	
N2	101 (26.0)	11 (23.4)	
N3	101 (26.0)	12 (25.5)	
Pathological TNM stage			0.712
I	78 (20.1)	10 (21.3)	
II	113 (29.1)	11 (23.4)	
III	197 (50.8)	26 (55.3)	

Bold: statistically significant.

As shown in **Table 2**, for both intestinal-type and diffuse-type GC, there was no significant difference between patients with cardia GC and those with noncardia GC in terms of clinicopathological features.

**Table 2:** Clinical profile between cardia and non-cardia GC patients in intestinal-type and diffuse-type GC.

Variables	Intestinal-type GC			Diffuse-type GC		
	Non-cardia	Cardia	*P* value	Non-cardia	Cardia	*P* value
	n = 203	n = 32		n = 185	n = 15	
	n (%)	n (%)		n (%)	n (%)	
Age (years old)			0.547			0.398
<65	68 (33.5)	9 (28.1)		90 (48.6)	9 (60.0)	
≧65	135 (66.5)	23 (71.9)		95 (51.4)	6 (40.0)	
Gender			0.174			0.220
Male	156 (76.8)	28 (87.5)		116 (62.7)	7 (46.7)	
Female	47 (23.2)	4 (12.5)		69 (37.3)	8 (53.3)	
Tumor size (cm)			0.694			0.282
<5	90 (44.3)	13 (40.6)		61 (33.0)	7 (46.7)	
≧5	113 (55.7)	19 (59.4)		124 (67.0)	8 (53.3)	
Extent of lymphadenectomy			0.846			0.960
D1+	60 (29.6)	10 (31.3)		38 (20.5)	3 (20.0)	
D2	143 (70.4)	22 (68.8)		147 (79.5)	12 (80.0)	
Gross appearance			0.524			0.579
Superficial type	30 (14.8)	4 (12.5)		28 (15.1)	2 (13.3)	
Borrmann types 1 & 2	62 (30.5)	13 (40.6)		40 (21.6)	5 (33.3)	
Borrmann types 3 & 4	111 (54.7)	15 (46.9)		117 (63.2)	8 (53.3)	
Adjuvant chemotherapy	20 (9.9)	5 (15.6)	0.325	34 (18.4)	4 (26.7)	0.431
Lymphovascular invasion	146 (71.9)	22 (68.8)	0.712	129 (69.7)	10 (66.7)	0.804
Pathological T category			0.429			0.943
T1	32 (15.8)	5 (15.6)		27 (14.6)	3 (20.0)	
T2	44 (21.7)	3 (9.4)		22 (11.9)	2 (13.3)	
T3	65 (32.0)	12 (37.5)		67 (36.2)	5 (33.3)	
T4	62 (30.5)	12 (37.5)		69 (37.3)	5 (33.3)	
Pathological N category			0.396			0.603
N0	79 (38.9)	9 (28.1)		45 (24.3)	3 (20.0)	
N1	29 (14.3)	8 (25.0)		33 (17.8)	4 (26.7)	
N2	53 (26.1)	9 (28.1)		48 (25.9)	2 (13.3)	
N3	42 (20.7)	6 (18.8)		59 (31.9)	6 (40.0)	
Pathological TNM stage			0.132			0.496
I	50 (24.6)	8 (25.0)		28 (15.1)	2 (13.3)	
II	65 (32.0)	5 (15.6)		48 (25.9)	6 (40.0)	
III	88 (43.3)	19 (59.4)		109 (58.9)	7 (46.7)	

### Initial Recurrence Patterns

Among the 435 patients, 145 (33.3%) experienced tumor recurrence. As shown in **Table 3**, no significant difference was observed in the initial recurrence pattern between cardia and noncardia GC or between intestinal-type GC and diffuse-type GC.

**Table 3:** The initial recurrence pattern between cardia and non-cardia GC patients in intestinal-type and diffuse-type GC.

Recurrence pattern	All GC			Intestinal-type GC			Diffuse-type GC		
	Non-cardia	Cardia	*P* value	Non-cardia	Cardia	*P* value	Non-cardia	Cardia	*P* value
	n = 388	n = 47		n = 242	n = 121		n = 185	n = 15	
	n (%)	n (%)		n (%)	n (%)		n (%)	n (%)	
Total patients with recurrence	129 (33.2)	16 (34.0)	0.913	63 (31.0)	9 (28.1)	0.740	66 (35.7)	7 (46.7)	0.395
Locoregional recurrence	56 (14.4)	3 (6.4)	0.128	27 (13.3)	3 (9.4)	0.536	29 (15.7)	0	0.134
Distant metastasis	110 (28.4)	15 (31.9)	0.610	56 (27.6)	8 (25.0)	0.760	54 (29.2)	7 (46.7)	0.157
Peritoneal dissemination	51 (13.1)	7 (14.9)	0.739	19 (9.4)	3 (9.4)	0.998	32 (17.3)	4 (26.7)	0.364
Hematogenous metastasis	58 (14.9)	7 (14.9)	0.992	35 (17.2)	5 (15.6)	0.821	23 (12.4)	2 (13.3)	0.919
Liver	36 (9.3)	7 (14.9)		24 (11.8)	5 (15.6)		12 (6.5)	2 (13.3)	
Lung	11 (2.8)	0		7 (3.4)	0	0.286	4 (2.2)	0	
Bone	11 (2.8)	1 (2.1)		6 (3.0)	1 (3.1)	0.958	5 (2.7)	0	
Brain	1 (0.3)	0		0			1 (0.5)	0	
Adrenal	2 (0.5)	1 (2.1)		0	1 (3.1)		2 (1.1)	0	
Skin	4 (1.0)	0		2 (1.0)	0		2 (1.1)	0	
Distant lymphatic recurrence	29 (7.5)	4 (8.5)	0.800	16 (7.9)	1 (3.1)	0.334	13 (7.0)	3 (20.0)	0.105
Virchow’s lymph node	6 (1.5)	0		4 (2.0)	0	0.423	2 (1.1)	0	
Paraaortic lymph node	25 (6.4)	4 (8.5)		13 (6.3)	1 (3.1)	0.466	12 (6.5)	3 (20.0)	

Some patients had more than one recurrence pattern.

### Survival Analysis

**Figure 1:**
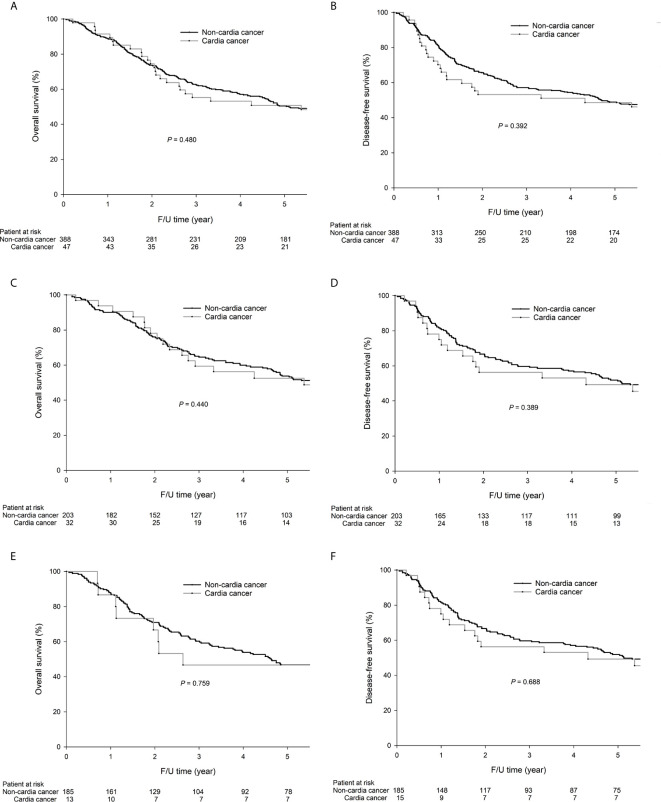
The 5-year OS (50.8% vs. 50.5%, *P* = 0.480) and DFS (48.6% vs. 48.9%, *P* = 0.392) rates were not significantly different between cardia and noncardia GC. For intestinal-type GC patients, the 5-year OS (53.8% vs. 52.5%, *P* = 0.440) and DFS (49.3% vs. 51.9%, *P* = 0.389) rates were not significantly different between cardia and noncardia GC. For diffuse-type GC patients, the 5-year OS (46.7% vs. 46.8%, *P* = 0.759) and DFS (46.7% vs. 45.4%, *P* = 0.688) rates were not significantly different between cardia and noncardia GC. The survival curves are shown as follows: **(A)** OS curves of all GC patients; **(B)** DFS curves of all patients; **(C)** OS curves of intestinal-type GC patients; **(D)** DFS curves of intestinal-type GC patients; **(E)** OS curves of diffuse-type GC patients; **(F)** DFS curves of diffuse-type GC patients.

As shown in **Figure 1**, there was no significant difference in the 5-year OS (50.8% vs. 50.5%, *P* = 0.480, **Figure 1A**) or DFS (48.6% vs. 48.9%, *P* = 0.392, **Figure 1B**) between cardia and noncardia GC patients. For intestinal-type GC, the 5-year OS (53.8% vs. 52.5%, *P* = 0.440, **Figure 1C**) and DFS (49.3% vs. 51.9%, *P* = 0.356, **Figure 1D**) rates were not significantly different between cardia and noncardia GC patients. For diffuse-type GC, the same trends were observed for 5-year OS (46.7% vs. 46.8%, *P* = 0.759, **Figure 1E**) and DFS (46.7% vs. 45.4%, *P* = 0.688, **Figure 1F**) between cardia and noncardia GC patients.

Univariable analysis demonstrated that age, sex, gross appearance, lymphovascular invasion, pathological T and N categories, and *PIK3CA* amplification were significantly associated with OS. The seven factors mentioned above, as well as tumor location (cardia GC vs noncardia GC), were included in the multivariable analysis. The Cox proportional hazard model demonstrated that age, gross appearance, and pathological T and N categories were independent prognostic factors of OS (**Table 4**).

**Table 4:** Univariate and multivariate analysis of factors affecting OS of GC patients after curative surgery.

Variables	Univariate analysis			Multivariate analysis		
	HR	95%CI	*P* value	HR	95%CI	*P* value
Age (years old)			**<0.001**			**<0.001**
<65	1.00			1.00		
≧65	1.78	1.373–2.308		1.93	1.456–2.550	
Gender			**<0.001**			0.173
Male	1.00			1.00		
Female	0.56	0.423–0.753		0.80	0.587–1.101	
Tumor location			0.480			0.819
Non-cardia	1.00			1.00		
Cardia	1.14	0.790–1.652		1.05	0.713–1.535	
Extent of lymphadenectomy			0.405			
D1+	1.00					
D2	0.89	0.688–1.163				
Gross appearance			**<0.001**			**0.005**
Superficial type	1.00			1.00		
Bormann types 1 & 2	2.23	1.402–3.561		1.66	0.975–2.813	
Bormann types 3 & 4	3.14	2.049–4.824		2.21	1.314–3.706	
Lymphovascular invasion			**<0.001**			0.337
Absent	1.00			1.00		
Present	2.55	1.869–3.476		1.21	0.822–1.772	
Lauren’s classification			0.399			
Intestinal type	1.00					
Diffuse type	1.11	0.873–1.409				
Adjuvant chemotherapy			0.752			
No	1.00					
Yes	1.06	0.737–1.527				
Pathological T category			**<0.001**			**0.007**
T1	1.00			1.00		
T2	1.62	0.959–2.729		0.74	0.395–1.369	
T3	2.43	1.543–3.823		0.86	0.475–1.539	
T4	4.13	2.667–6.408		1.28	0.710–2.317	
Pathological N category			**<0.001**			**<0.001**
N0	1.00			1.00		
N1	0.93	0.606–1.431		0.85	0.540–1.329	
N2	1.98	1.412–2.771		1.49	1.031–2.147	
N3	5.22	3.765–7.237		4.30	2.956–6.245	
MSI status			0.962			
MSI-L/S	1.00					
MSI-H	1.01	0.659–1.550				
*PIK3CA* amplification			**0.043**			0.131
Absent	1.00			1.00		
Present	1.28	1.008–1.626		1.21	0.945–1.549	
*PI3K/AKT* pathway mutation			0.669			
Absent	1.00					
Present	0.93	0.679–1.282				
*TP53* mutation			0.096			
Absent	1.00					
Present	1.32	0.952–1.827				
*ARID1A* mutation			0.235			
Absent	1.00					
Present	1.25	0.864–1.813				

OS, overall survival; DFS, disease-free survival.

Bold: statistically significant.

### Genetic Analysis

As shown in **Table 5**, for intestinal-type GC, patients with cardia GC had increased *PIK3CA* amplification (59.4% vs. 38.4%, P = 0.025) compared to patients with noncardia GC. For diffuse-type GC, patients with cardia GC had increased HER2 expression (26.7% vs. 4.9%) compared to patients with noncardia GC.

**Table 5:** The molecular differences between cardia and non-cardia GC patients after curative surgery.

Variables	All GC			Intestinal-type GC			Diffuse-type GC		
	Non-cardia	Cardia	*P* value	Non-cardia	Cardia	*P* value	Non-cardia	Cardia	*P* value
	n = 388	n = 47		n = 203	n = 32		n = 185	n = 15	
	n (%)	n (%)		n (%)	n (%)		n (%)	n (%)	
MSI status			0.231			0.516			0.220
MSI-L/S	354 (90.5)	45 (95.7)		183 (90.1)	30 (93.8)		168 (90.8)	15 (100.0)	
MSI-H	37 (9.5)	2 (4.3)		20 (9.9)	2 (6.3)		17 (9.2)	0	
HP infection	143 (36.9)	14 (29.8)	0.341	68 (33.5)	10 (31.3)	0.802	75 (40.5)	4 (26.7)	0.290
EBV infection	35 (9.0)	6 (12.8)	0.407	18 (8.9)	5 (15.6)	0.232	17 (9.2)	1 (6.7)	0.743
*PIK3CA* amplification	180 (46.4)	26 (55.3)	0.247	78 (38.4)	19 (59.4)	**0.025**	102 (55.1)	7 (46.7)	0.526
*HER2* expression	37 (9.5)	7 (14.9)	0.250	28 (13.8)	3 (9.4)	0.492	9 (4.9)	4 (26.7)	**0.001**
Genetic mutations									
*PI3K/AKT* pathway	65 (16.8)	5 (10.6)	0.281	48 (23.6)	3 (9.4)	0.069	17 (9.2)	2 (13.3)	0.599
*TP53*	54 (13.9)	5 (10.6)	0.535	28 (13.8)	3 (9.4)	0.492	26 (14.1)	2 (13.3)	0.938
*ARID1A*	45 (11.6)	7 (14.9)	0.511	29 (14.3)	5 (15.6)	0.841	16 (8.6)	2 (13.3)	0.542
*BRAF*	2 (0.5)	0	0.622	2 (1.0)	0	0.573	0	0	–
*KRAS*	10 (2.6)	0	0.266	6 (3.0)	0	0.325	4 (2.2)	0	0.565

MSI, microsatellite instability; MSI-H, MSI-high; MSI-L/S, MSI-low/stable; EBV, Epstein–Barr virus.

Bold: statistically significant.

## Discussion

Cardia GC patients have been reported to have a more advanced tumor stage and unfavorable clinicopathological features at diagnosis than noncardia GC patients (3). However, the 5-year OS was shown to be similar between cardia and noncardia GC patients at each TNM stage (3). In the present study, the clinicopathological features, recurrence patterns, and prognoses were similar between cardia and noncardia GC patients; however, more intestinal-type tumors were observed in cardia GC patients. The multivariable analysis also demonstrated that cardia GC itself is not an independent prognostic factor. According to our results and those of other studies (3), late-stage diagnosis is the main cause of the poorer patient prognosis for cardia GC than noncardia GC.

*HER2* expression is higher in the gastric cardia than in other locations of the stomach (6). The correlation between *HER2* expression and Lauren’s histologic types remains controversial. Some studies have reported that *HER2* expression was associated with intestinal-type GC (7); however, others have reported that *HER2* expression was correlated with diffuse-type GC (8). In addition, the presence of HP infection may induce *HER2* overexpression (17). Consequently, the discrepancy among studies might be due to the small sample sizes and differences in racial and environmental factors. One of the novel findings of the present study is that *HER2* expression is significantly higher in cardia GC patients than in noncardia GC patients among those with diffuse-type GC but not intestinal-type GC. A randomized controlled trial demonstrated that trastuzumab in combination with chemotherapy improved survival in patients with advanced gastric or gastroesophageal junction cancer compared with chemotherapy alone (16). The frequency of *HER2* expression in the GC patients enrolled in the present study was 10.1%, which was considered the cutoff percentage for targeted therapy benefits. According to our results, we recommend performing IHC staining for *HER2* in patients with diffuse-type GC, as targeted therapy may be beneficial for this subtype.

It has been reported that *PIK3CA* amplification was associated with GC-related death (18); in addition, patients with *PIK3CA* amplification had worse survival than patients without *PIK3CA* amplification, which is similar to our results. In the present study, cardia GC was associated with higher *PIK3CA* amplification than noncardia GC but only among intestinal-type GC patients, not diffuse-type GC patients. *In vivo* and *in vitro* studies demonstrated the significance of proapoptotic and antiproliferative inhibition of *PI3K*. Therapeutic agents targeting *PI3K* have been applied in phase 1 and phase 2 clinical trials among patients with gastrointestinal cancer. In addition, combination therapy with *PI3K* inhibitors and other targeted therapies are under investigation (19). The frequency of *PIK3CA* amplifications in the enrolled GC patients in the present study was 47.4%, which was considered the cutoff percentage for targeted therapy benefits. According to our results, we recommend testing for *PIK3CA* amplification in intestinal-type cardia cancer patients, as they may benefit from targeted therapy.

Four molecular subtypes of GC using the database of The Cancer Genome Atlas (TCGA) have been reported (20); however, the etiology and genetic alterations of cardia GC are still not well known. Our results demonstrated that cardia GC presented with more intestinal-type tumors than noncardia GC, which was also reported in a TCGA analysis (20). The differences between cardia GC and noncardia GC are multifactorial, involving etiology, biological behaviors, lifestyle, HP infection, EBV infection, gastroesophageal reflux disease, environment, and genetic and epigenetics (21). For economic reasons, we used the MassARRAY method to analyze genetic mutations. Despite the high cost, we believe that next-generation sequencing could provide additional information on differences in the genetic alterations between cardia and noncardia GC; we plan to implement this technique in future research on this topic.

In the present study, the top three genetic mutations in gastric cardia and noncardia cancer were in the *PI3K/AKT* pathway, *TP53* and *ARID1A*. In the present study, the frequency of *PI3K*/*AKT* pathway mutations was not significantly different between patients with cardia and noncardia GC. It has been reported that only tumors located in the middle third of the stomach show an increase in *PIK3CA* mutations (22). EBV infection was associated with *PIK3CA* mutation, especially in the body of the stomach (20). EBV infection and *PIK3CA* mutation are more likely to induce carcinogenesis in the middle third of the stomach. *TP53* mutations can upregulate the transcription of vascular endothelial growth factor receptor-2 (VEGFR2) by promoter remodeling (23). A significant increase in VEGF expression was observed in cancer patients with *TP53* mutations (24). The anti-VEGFR2 inhibitor ramucirumab combined with paclitaxel has been approved as the standard second-line systemic therapy (25); however, the survival benefit is limited and novel combination therapies with other targeted therapies and immunotherapy may be beneficial for patient outcome (26). *ARID1A*, a tumor suppressor gene, has been identified as the second most mutated gene after *TP53* in GC (19), and *ARID1A* deficiency is associated with poor prognosis and lymph node metastasis in GC patients (27). Loss of *ARID1A* expression correlated with increased PD-L1 expression (28), and *ARID1A* mutation was associated with MSI and EBV infection (29). Consequently, immunotherapy might be beneficial for GC patients with *ARID1A* alterations.

One of the limitations of this study is its retrospective and single-center nature. In addition, the number of cardia GC patients in the molecular analysis was small, which may have caused selection bias. In the future, more patients from different countries and races are needed to validate our results, which may provide convincing evidence for GC treatment in the future.

## Conclusion

In conclusion, cardia GC is not an independent prognostic factor. For intestinal-type GC, *PIK3CA* amplification was more common in cardia GC patients than in noncardia GC patients. For diffuse-type GC, *HER2* expression was more common in cardia GC patients than in noncardia GC patients. Targeted therapy may be beneficial for these patient subgroups.

## Data Availability Statement

The original contributions presented in the study are included in the article/supplementary material. Further inquiries can be directed to the corresponding author.

## Ethics Statement

The studies involving human participants were reviewed and approved by The Institutional Review Board of Taipei Veterans General Hospital. The patients/participants provided their written informed consent to participate in this study.

## Author Contributions

Conceptualization: S-MP, K-HH, M-HC, W-LF, YC, S-SL, AF-Y, C-WW, and Y-MS. Data curation: S-MP and W-LF. Formal analysis: S-MP and W-LF. Funding acquisition: W-LF. Investigation: K-HH, and W-LF. Methodology: K-HH, and W-LF. Writing—original draft: S-MP and W-LF. Writing—review and editing: W-LF. All authors contributed to the article and approved the submitted version.

## Funding

This study was supported by research grants from the Ministry of Science and Technology, Taiwan (107-2314-B-075-007, 107-2314-B-075-005-MY2) and Taipei Veterans General Hospital (V107C-040, V109C-105). The funding sources had no role in the study design, data analysis, or writing and submission of the manuscript.

## Conflict of Interest

The authors declare that the research was conducted in the absence of any commercial or financial relationships that could be construed as a potential conflict of interest.

## Abbreviations

AJCC, American Joint Committee on Cancer; DFS, Disease-free survival; EBV, Epstein–Barr virus; FU, Fluorouracil; GC, Gastric cancer; HP, *Helicobacter pylori*; IHC, Immunohistochemical; ISH, *In situ* hybridization; MSI, Microsatellite instability; MSI-H, Microsatellite instability-high; MSI-L/S, Microsatellite instability-low/stable; OS, Overall survival; PCR, Polymerase chain reaction; SNP, Single nucleotide polymorphism; TCGA, The Cancer Genome Atlas; TNM, Tumor, node, metastasis; UICC, Union for International Cancer Control; VEGFR2, Vascular endothelial growth factor receptor-2.
